# Bronchoscopic visualization of a cavity in entirety: an unusual finding

**DOI:** 10.1002/rcr2.649

**Published:** 2020-08-31

**Authors:** Deebya Raj Mishra, Narendra Bhatta, Achyut Bhakta Acharya, Avatar Verma, Rejina Shahi, Niharika Shah

**Affiliations:** ^1^ Department of Pulmonary, Critical Care and Sleep Medicine BP Koirala Institute of Health Sciences Dharan Nepal; ^2^ Department of Pathology BP Koirala Institute of Health Sciences Dharan Nepal

**Keywords:** Bronchoscopy, cavity

## Abstract

It is unusual to be able to visualize an entire cavity with such clarity.

## Graphical abstract

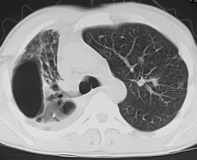

## Video

**Figure 1:**
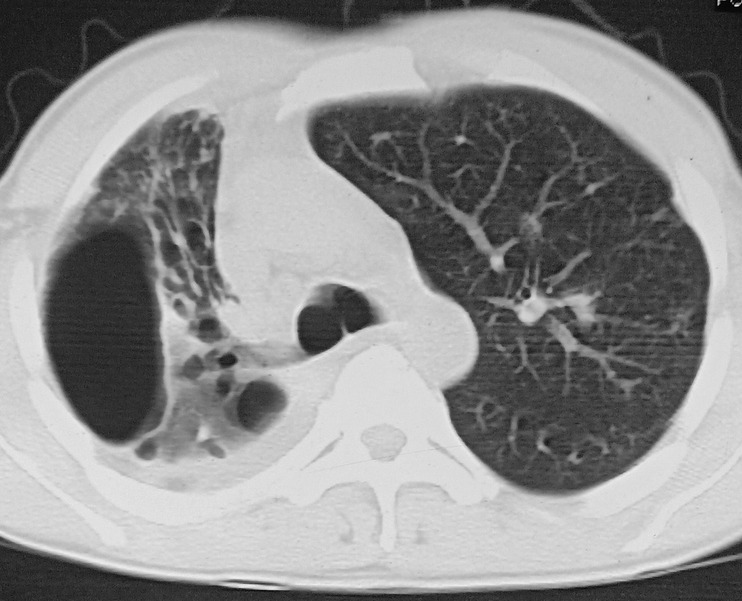
Computed tomography (CT) of the chest showing cavity and fibro‐bronchiectatic changes in the right upper lobe.

A 38‐year**‐**old male, with a history of sputum‐positive pulmonary tuberculosis treated seven years back, presented with occasional haemoptysis of six months duration. Computed tomography of the chest showed a large cavity with fibro‐bronchiectatic changes in the right upper lobe (Fig. 1). During flexible bronchoscopy, the conventional scope (Pentax EB15‐J10, Hoya, Japan, outer diameter 5.4 mm) could be negotiated through the opening of apical segmental bronchus of right upper lobe which opened into the inside of a large cavity. The inside of the entire cavity could be visualized with ease and appeared epithelialized (Video S1, Supporting Information). Haemoptysis was attributed to bronchiectatic changes.

### Disclosure Statement

Appropriate written informed consent was obtained for publication of this case report and accompanying images.

## Supporting information


**Video S1.** Bronchoscopy showing the localization of the cavity in the apical segment of right upper lobe and the inside of the cavity

